# Supplementary material for: Modulating Expression of Thioredoxin Interacting Protein (TXNIP) Prevents Secondary Damage and Preserves Visual Function in a Mouse Model of Ischemia/Reperfusion
Source: Int J Mol Sci. 2019 Aug 15;20(16):3969. doi: 10.3390/ijms20163969 (PMC6721134; doi:10.3390/ijms20163969)
Supplement: Supplementary file 1 [file ijms-20-03969-s001.pdf]

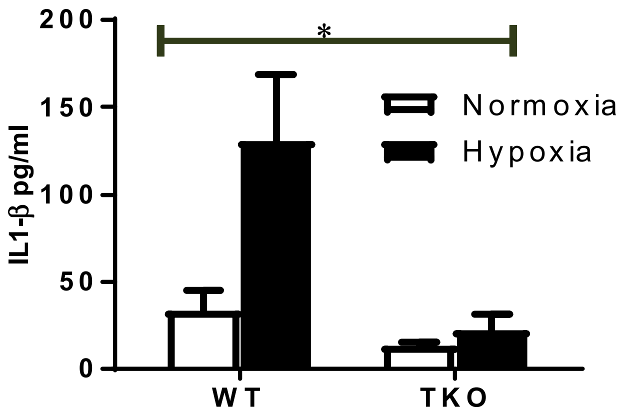


**Figure-S1.** Statistical analysis of secreted IL-1β protein levels assessed by ELISA from primary retinal Muller cells isolated from wild type (WT) and TXNIP knockout (TKO) mice. Results showed that WT-cells subjected to hypoxia showed significant activation of TXNIP-NLRP3 inflammasome activation evident by release of cleaved IL-1β compared to normoxia. (n=5-6,* $P<0.05$, two-way ANOVA showed significant effect of hypoxia and gene deletion).

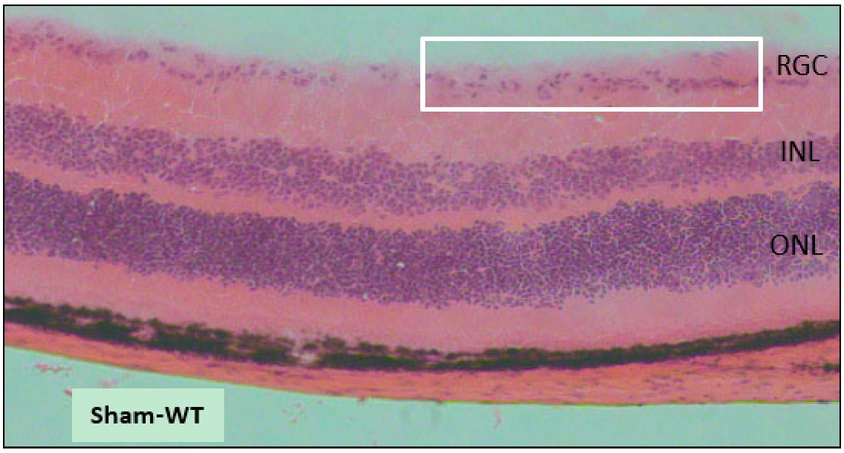


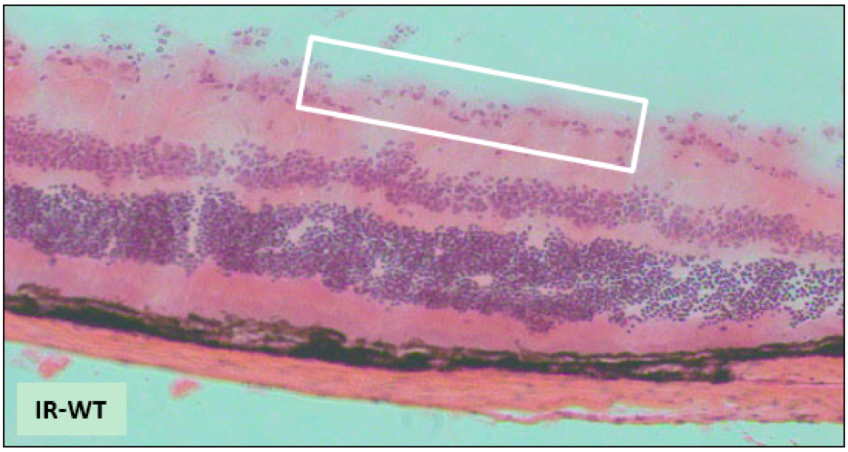

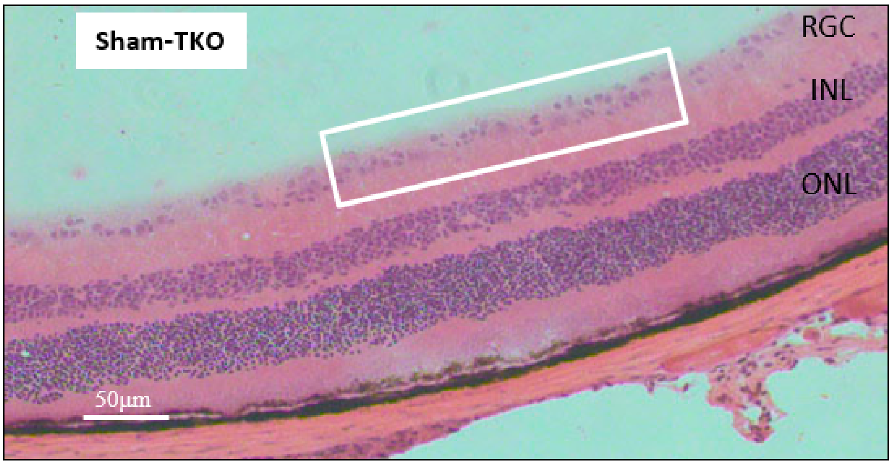


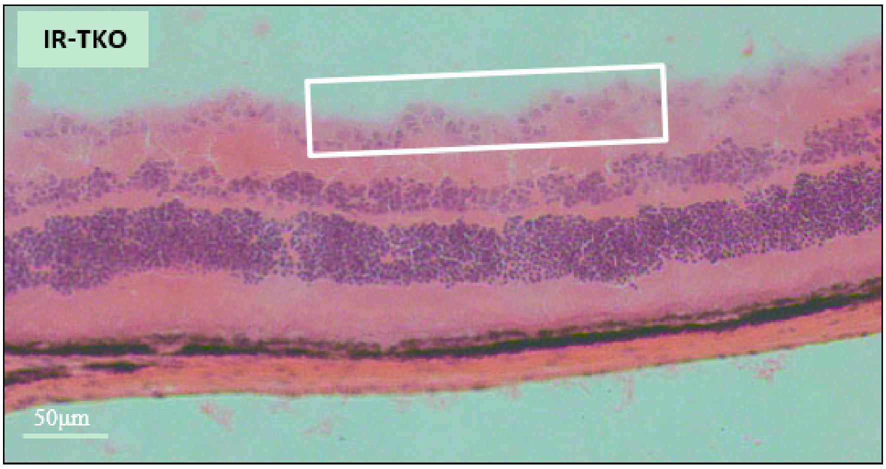


**Figure-S2.** Representative images of whole retina section showing that IR caused significant reduction in number of cells in ganglion cell layer in the peripheral retinas of the WT, but not of TKO mice when compared to shams after 3-days. Note that the white boxed-window is shown enlarged as a representative in Fig.6A.

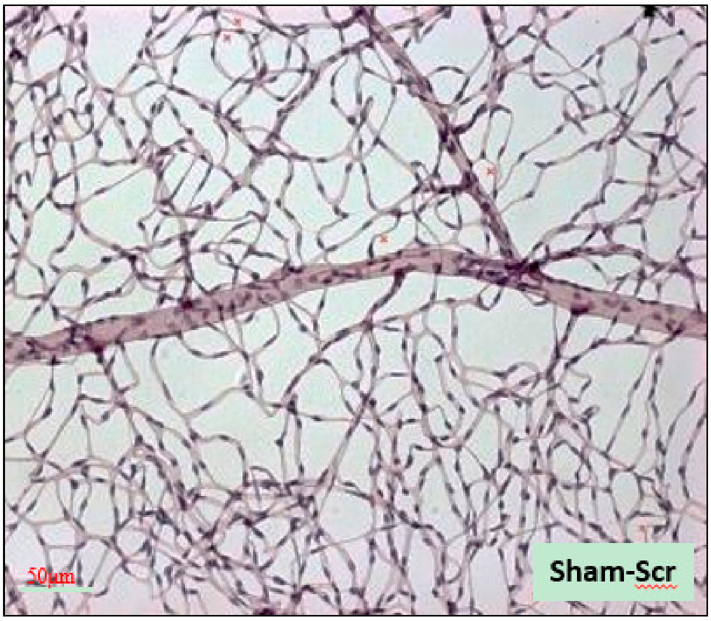
50μm
Sham-Scr

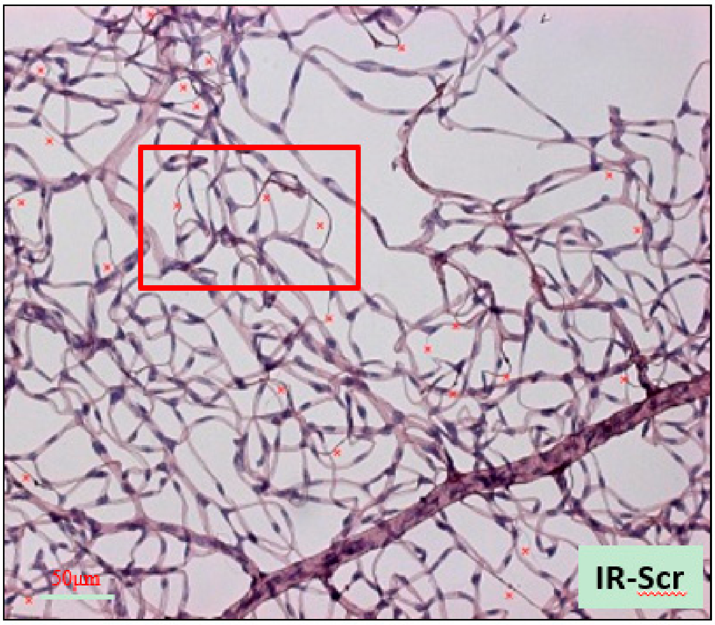
50μm
IR-Scr

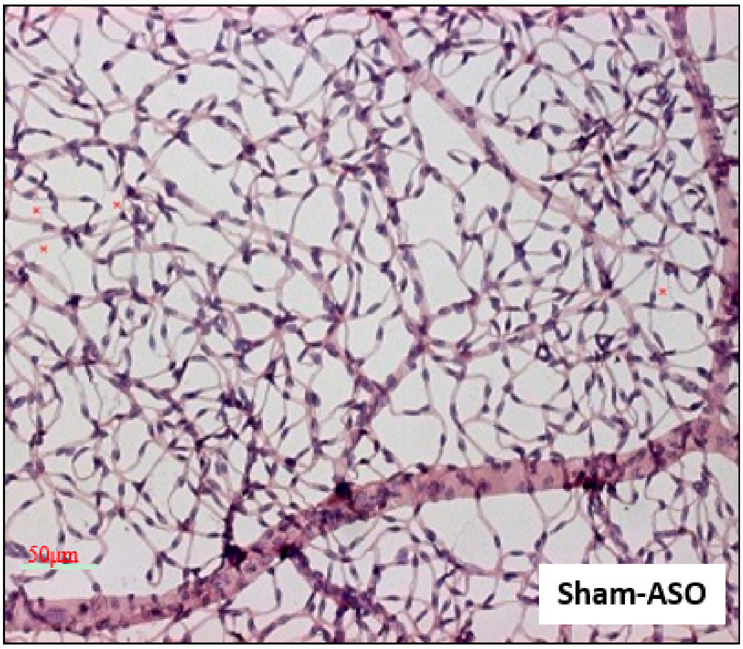


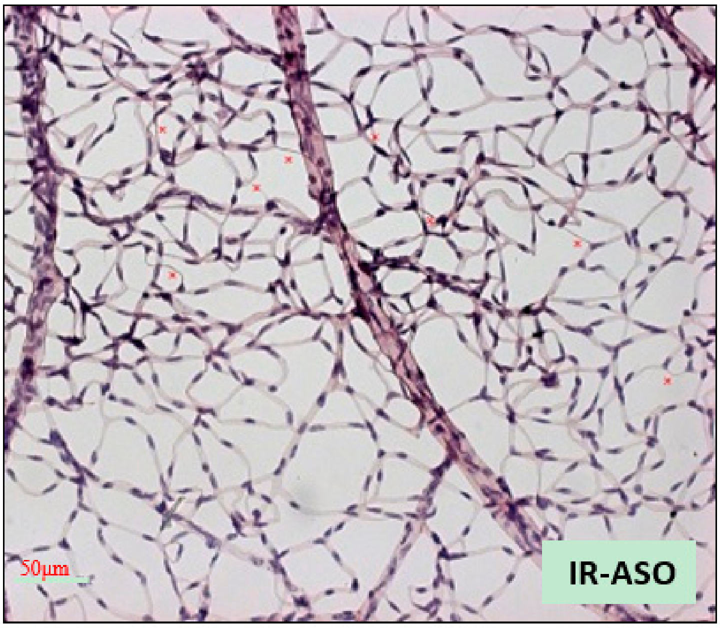


**Figure-S3. R**epresentative images showing that transient ischemia increased number of acellular capillaries (marked with red x), which was reduced by inhibiting TXNIP expression 2 days after IR. Note that the red window is shown enlarged as a representative in Fig.8A.
